# Bluetongue Virus Infection in Farm Dogs Exposed to an Infected Sheep Flock in South Africa

**DOI:** 10.1155/2024/2446398

**Published:** 2024-07-29

**Authors:** Josef Hanekom, Karen Ebersohn, Lisa Penzhorn, Melvyn Quan, Andrew Leisewitz, Alan Guthrie, Geoffrey T. Fosgate

**Affiliations:** ^1^ Companion Animal Clinical Studies University of Pretoria Faculty of Veterinary Science, Onderstepoort, Pretoria, South Africa; ^2^ Department of Tropical Diseases University of Pretoria Faculty of Veterinary Science, Onderstepoort, Pretoria, South Africa; ^3^ Equine Research Center University of Pretoria Faculty of Veterinary Science, Onderstepoort, Pretoria, South Africa; ^4^ University of Pretoria Faculty of Veterinary Science, Onderstepoort, Pretoria, South Africa; ^5^ Production Animal Studies University of Pretoria Faculty of Veterinary Science, Onderstepoort, Pretoria, South Africa

## Abstract

In 2021, a pregnant Rottweiler dog living on a sheep farm was diagnosed with clinical bluetongue (BT) infection. This study reports on the investigation of this farm where bluetongue virus (BTV) infection was diagnosed in this atypical host species. Samples were collected during farm visits 14, 28, 60, and 89 days after the onset of clinical signs in the pregnant Rottweiler. Blood was collected from all farm dogs (*n* = 6) and tested for BTV genome using a reverse-transcriptase quantitative PCR (RT-qPCR) assay and BTV antibodies with the competitive ELISA (cELISA) and dogs positive by RT-qPCR were further tested using virus neutralization (VN) serological testing. Blood was also collected from 16 sick sheep and tested using RT-qPCR. Midges were trapped on the study farm using an Onderstepoort UV light trap placed above a sheep pen for 36 hr at the first farm (14 days) visit. Parous/gravid midges were tested by BTV RT-qPCR in batches of up to 200 midges per species. Blood-fed midges (*n* = 308) were tested using a PCR species probe (KAPA Multiplex Master Mix) to identify the host species on which the midge had fed. Three dogs (*n* = 3/6) had detectable BTV RNA with RT-qPCR and high VN antibody titers to BTV. All RT-qPCR-positive dogs and one additional dog tested cELISA seropositive (*n* = 4/6). Bluetongue virus RNA was detected in 5/16 sheep tested. The most abundant midge species was *Culicoides imicola* (99.3%) and BTV was only detected in this species (*n* = 3/4 batches of 200 parous midges). Dog blood was not detected in any blood-fed midges tested. The occurrence of natural BT viraemia in exposed dogs creates a potential risk of BTV entry into BT-free countries through dog importation. It remains unclear whether BT viremia in dogs is capable of onward transmission.

## 1. Introduction

Bluetongue (BT) is a midge-borne disease of ruminants caused by infection with the double-stranded RNA bluetongue virus (BTV) (family *Sedoreoviridae*, genus *Orbivirus*) with 28 recognized serotypes [1, 2, 3]. Bluetongue virus infection typically causes mild disease or subclinical infections in wild ruminants, cattle, and goats, while sheep tend to suffer the highest mortalities (average 2%–30%) [2, 4]. The most important transmission route for BTV is through *Culicoides* midges, which are integral to the distribution, transmission, and persistence of BTV [5, 6]. The importance of each vector species varies geographically with *Culicoides imicola* and *Culicoides bolitinos* considered the most important BTV vector species in South Africa [7, 8]. Bluetongue is a World Organization for Animal Health (WOAH) listed disease [3] with outbreaks in previously BTV-free countries have devastating economic impacts as evidenced by the 2008 outbreak in Europe [9, 10, 11].

Although multiple carnivorous species develop antibodies to BTV [12], only the European lynx (*Lynx lynx*) [13] and domestic dogs (*Canis lupus familiaris*) have reportedly died from BT [14, 15]. Several pregnant dogs aborted, developed respiratory distress, and died acutely after receiving modified live dog vaccines contaminated with BTV serotype 11 (BTV-11) in the USA in 1992–1993 [14, 15, 16, 17]. Subsequently, BTV-11 was isolated in aborted dog foetuses with natural transmission suspected to be by midge-borne transmission or iatrogenic by venereal transmission during artificial insemination of the pregnant dogs [18, 19, 20]. Recently another two pregnant dogs have been reported to have developed BT with natural transmission, one in South Africa in 2021 (included in this study) [21] and another in the Netherlands in 2023 [22].

Population studies of domestic dogs have shown a surprisingly high BTV seroprevalence (20%–21%) in African countries [12, 23]. It was previously suggested that subclinically viraemic dogs might have been the source of some BT outbreaks in Europe [12, 24]. However, naturally occurring BT viraemia in subclinical dogs has not been previously reported. The route of BTV transmission to dogs is suspected to be either through ingestion of BTV-contaminated meat [12] or through midge-borne transmission [24]. Despite a high seroprevalence, few cases of BT in dogs are reported suggesting that dogs rarely develop clinical disease following exposure.

This study describes an investigation that followed a diagnosis of clinical BT in a domestic dog from a sheep farm in South Africa. As both natural transmission and clinical disease in a dog were not typical of BT, this investigation was conducted to better understand the context of the infection. The primary objective of this investigation was to describe the exposure to and infection with BTV in dogs, sheep, and midges on the affected sheep farm. A secondary objective was to describe the midge species and their feeding habits on this farm.

## 2. Materials and Methods

### 2.1. Background and Study Area

This study was a prospective observational field investigation focusing on dogs living on a sheep farm where a dog was diagnosed with BT disease. The study farm was located on the border of the Gauteng and North-West Province (25°45′00″S 27°56′36″E), South Africa, where six dogs, two horses, and approximately 400 Dorper (meat breed) sheep were kept. The study period coincided with the end of the lambing season and the area had recently experienced heavy rainfall due to a tropical storm Eloise.

On 25 February 2021 (defined as Day 0), a 3-year-old pregnant Rottweiler dog (Dog 1) living on the study farm developed clinical signs of BT. The dog was presented to the Onderstepoort Veterinary Academic Hospital (OVAH), Pretoria, South Africa, in severe respiratory distress. The dog survived with symptomatic treatment but subsequently aborted the puppies. Samples submitted while clinical signs (Day 3) were present in the dog tested positive using a pan-BTV real-time reverse transcription polymerase chain reaction assay (RT-qPCR) (*Ct* value of 24.7). The clinical findings and medical management of this dog have been reported elsewhere [21].

All sample size calculations in this study were performed using open source software (Sergeant, ESG, 2018. Epitools Epidemiological Calculators. Ausvet. Available at: http://epitools.ausvet.com.au).

### 2.2. Sample Selection and Study Population

#### 2.2.1. Dogs

**Figure 1:**
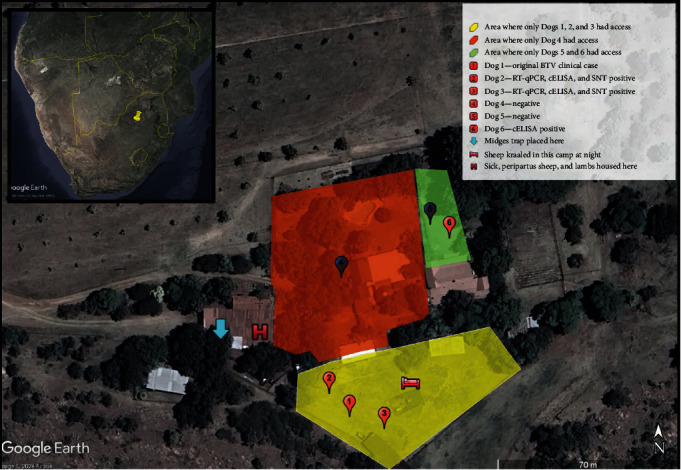
Map of the farm that had a BT infection involving sheep and dogs. Small map insert with yellow pin shows the location of the farm on a map of Africa. Due to interdog aggression, dogs were kept apart and the location where the dogs (*n* = 6) were kept is shown (numbered pins). Dogs 1, 2, and 3 were kept in a camp where sheep were penned at night (denoted by red bed), while Dogs 4, 5, and 6 had no direct access to sheep. Ill and recently lambed sheep were housed in a separate observation pen (denoted red H) in which midges were trapped (blue arrow). Maps are made on Google Earth Pro (version 10.49.0.0, 2023, Google LLC).

The six dogs that lived on the farm were kept for security with occasional natural breeding (Table 1). Only Dog 1 was pregnant during this study. All dogs had a good body condition score and were provided with routine preventative medicines and annual vaccinations, the last of which was 4–5 months before the study onset. Preventive medications included recent administration of fluralaner (Bravecto®, MSD Animal Health) for ectoparasitic control. Aside from Dog 1, all other dogs were reported to be in good health besides one recovering from a femur fracture (Dog 4). The dogs were fed commercial dry pellet dog food and purposefully not fed any sheep products to prevent the dogs from “developing a taste for sheep.” Groups of dogs were permanently separated due to interdog aggression. Three dogs (Dogs 1, 2, and 3) were kept in the sheep enclosure with direct contact with sheep at night. The other three dogs (Dogs 4, 5, and 6) had no direct access to sheep or the other dogs (Figure 1).

Samples were collected during farm visits on Days 14, 28, 60, and 89. Blood was collected from all farm dogs (*n* = 6) including the original pregnant dog diagnosed with BT. Samples from all dogs were tested using RT-qPCR assays for BTV RNA and a competitive ELISA (cELISA) assay for BTV antibodies. Samples from dogs that tested positive for BTV RNA on RT-qPCR were additionally tested using virus neutralization (VN) for BTV antibody serotype determination.

#### 2.2.2. Sheep

Approximately 400 Dorper sheep (meat breed) were kept on the farm and were generally in poor body condition (body condition score 2/5). Sheep were kept in an enclosed camp close to the house at night with three dogs (Dogs 1, 2, and 3) to prevent stock theft. No sheep had been vaccinated for BTV for at least 2 years prior to the investigation. The farmer had experienced high mortality in his sheep flock and estimated that 20–30 sheep had died in the preceding months, but this was not investigated and there were no records of mortality or morbidity.

The samples size based on a freedom of disease calculation required 16 sheep to detect BTV in the sheep flock (20% prevalence, 95% confidence with 100% test accuracy). The selection was targeted for increased sensitivity of detection by selecting these 16 sheep from a “sick pen” that housed sick sheep, postpartum sheep, and lambs (Figure 1). Whole blood was collected from sheep and tested using the RT-qPCR assay for BTV RNA on the first farm visit (Day 14).

#### 2.2.3. *Culicoides* Midges

Midges were trapped using an ultraviolet light trap (an Onderstepoort downdraft light trap, 8 W, 220 V, 23 cm UV light downdraught) on the first farm visit (Day 14). The trap was active for approximately 36 hr and placed directly above sheep in the “sick pen” (Figure 1). Insects caught in the light traps were collected into a 500 ml plastic beaker containing 100 ml water and a 0.5% chlorhexidine (Savlon®) solution. The midges were filtered using a fine gauze filter, placed in 80% alcohol and sealed in a 250 ml plastic bottle. Midges were stored at 4°C until sorted within a week of collection.

A required sample size of 299 midge samples to detect canine blood meals was based on a freedom of disease calculation (1% prevalence, 95% confidence with 100% test accuracy). All available sorted blood-fed midges were selected (*n* = 240) with 40 tested individually, and the remaining in batches of 10 midges (*n* = 20 tests). An additional two batches of unsorted midges (1/24th of the total sample each) containing approximately 32 blood-fed midges were tested. In total, an estimated 308 blood-fed midges were tested with the PCR species probes for domestic animals (KAPA Multiplex Master Mix).

For the detection of BTV in parous/gravid midges, samples were separated into individual species and tested for BTV RNA with the RT-qPCR assay in batches of up to 200 midges per batch or all available midges (<200 available).

### 2.3. Laboratory Analysis and Sample Handling

#### 2.3.1. Sample Handling

Blood was collected from dogs and sheep by venipuncture of the cephalic or jugular veins by a veterinarian. At each collection, whole blood was collected in ethylenediaminetetraacetic acid (EDTA; BD Vacutainer® spray-coated KEDTA tubes), heparin (BD Vacutainer® spray-coated lithium heparin tubes), and serum (BD Vacutainer® Plus plastic serum tubes) tubes. Whole blood was frozen directly in the vacutainers while serum was separated into individually labeled serum tubes. The samples were initially frozen at −20°C, moved to a −80°C biobank freezer 6 months after collection, and transported on ice for laboratory analysis 8–12 months after sample collection.

#### 2.3.2. Real-Time BTV RT-qPCR

Nucleic acid detection was performed using a pan-BTV RT-qPCR according to the technique described by Hoffman et al. [25] using WOAH-recommended segment 10 (NS3) primers and probe [26] and Vetmax reagents (Life Technologies). Extraction of RNA was performed using the MagNPure 96™ Nucleic Acid Purification Kit (Applied Biosystems) following the manufacturer's protocol. Samples were classified as per laboratory recommendations as BTV positive when nucleic acid detection occurred below 36 cycle threshold (*Ct*), weak positive at between 37 and 40 cycle thresholds, and not detected at greater than 40 cycles.

#### 2.3.3. Competitive ELISA (cELISA)

A commercial competitive BTV VP7 iGG ELISA (Bluetongue Virus Antibody Test Kit, cELISA v2, Veterinary Medical Research and Development Laboratory, USDA Product Code 5010.20, Pullman WA, USA) was performed as per the manufacturer's instructions on dog sera samples. Reactions were assessed using a spectrophotometer with a reading wavelength of 630 nm and the percentage of inhibition (PI) was calculated as PI = 100 (1 − (sample OD/negative control OD)). Samples were considered positive when the PI was equal to or greater than 60.

#### 2.3.4. Virus Neutralization Serology

VN was performed as per standard methods using BTV stock virus (BTV-1–BTV-24) to a maximal dilution of 1 : 320. After inactivation of the serum, twofold serial dilutions were made in minimum essential medium (MEM) containing 5% foetal calf serum and 1 mg/ml Gentamycin. Equal volumes of virus, at a concentration of 100 TCID50, were added to all the wells, and the plates were incubated for 1 hr at 37°C in a humid atmosphere of 5% CO_2_ in air. An aliquot of 80 *µ*l of a Vero cell suspension, containing 480,000 cells/ml, was added to all the wells and the plates were incubated at 37°C in a humid atmosphere of 5% CO_2_ in air for 4–7 days. Titers were determined as the reciprocal of the highest serum dilution that showed 50% protection of the cells.

#### 2.3.5. Viral Isolation (VI)

The method employed for viral isolation was similar to that described by Spedicato et al. [27]. Confluent monolayers of BSR-T7 cells were prepared in 25 cm^2^ cell culture flasks (TPP®). Eagles' medium (Biowest®) containing 5% foetal bovine serum (Gibco®, ThermoFisher Scientific) was used to propagate cells. Whole blood samples in EDTA were inoculated onto cell monolayers and left to adsorb at 37°C for 1 hr. Cells were then washed with phosphate-buffered saline (PBS) and fresh medium containing 2% foetal bovine serum was added. Culture flasks were incubated at 37°C and cells were observed daily for evidence of cytopathic effect (CPE). When no CPE was observed after 14 days, cells were frozen and thawed, after which 0.5 ml was passaged onto freshly prepared cell monolayers, and the flasks were incubated and observed daily. This passage process was performed an additional three consecutive times prior to classification as negative.

#### 2.3.6. Midge Speciation

Trapped midges were divided into 24 approximately equal batches. Midges from one batch were individually identified and sorted according to species level, sex, and parity using identification keys [28, 29, 30] under a stereoscopic microscope. From the remaining batches, freshly blood-fed midges (*n* = 240) were identified, separated, and stored as individual midges (*n* = 40) and in batches of 10 midges (*n* = 20). These were kept in separately labeled Eppendorf tubes containing 80% ethanol and stored at 4°C until analysis.

#### 2.3.7. Midge Blood-Meal Species with PCR Species Probe

The protocol used for mammalian blood meal analysis had been described previously [31]. Briefly, midge samples were transferred manually into Eppendorf tubes containing 150 *µ*l of phosphate-buffered saline (PBS) and homogenized using a DWK Life Sciences Kimble™ and macerated in a biohazard flow hood. Nucleic acid was purified using the MagMAX™ CORE Nucleic Acid Purification Kit (Applied Biosystems) following the manufacturer's protocol. A total of 1 *µ*l of the purified nucleic acid solution was then added to 2.8 *µ*l of primer mix (including three universal primers and two primers for each of the following species: cat, dog, cow, horse, human, donkey, sheep, pig, and goat DNA), 1.2 *µ*l of water, and 5 *µ*l of KAPA Multiplex Master Mix was amplified by PCR. A total of 1 *µ*l of the amplified solution was then added to 9 *µ*l of HiDI Formamide + 0.25 *µ*l of Genescan 500 LIZ size standard and denatured. Initial denaturation was at 95°C for 3 min followed by 30 cycles of 95°C for 15 s, 60°C for 30 s, and 72°C for 30 s. The final extension occurred at 72°C for 10 min and was then cooled to 4°C. The final product was run on a 3500xl Sequencer—Thermo Fisher Scientific, utilizing a fragment analysis protocol with a 50 cm capillary and POP-7 TM polymer. Data files were transferred to STRand software for analysis.

## 3. Results

### 3.1. Dogs

Nucleic acid from the BTV was detected in three (50%) of the farm dogs. All positive dogs, Dog 1 (*Ct* 24.7), Dog 2 (*Ct* 36.0), and Dog 3 (*Ct* 39.1 classified as a weak positive) had direct contact with sheep (Figure 1 and Table 2). Dog 1 tested positive twice, during clinical disease (*Ct* 24.7) and at Day 28 (*Ct* 38.0).

**Figure 2:**
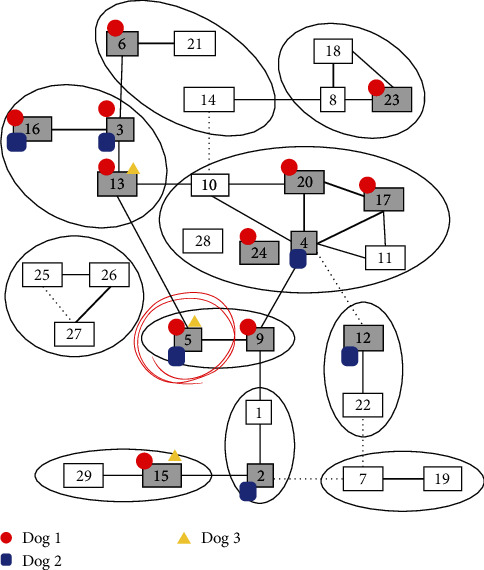
The SNT serotype of BTV antibodies that developed (>1 : 112 titers) in dogs that tested positive for BTV on real-time RT-qPCR. The serotypes found are overlayed (gray highlighted) onto a figure by van Rijn et al. (2021) showing the cross-neutralization of serotypes in sheep and cattle. The serotypes found are marked for Dog 1 (red circle), Dog 2 (yellow triangle), and Dog 3 (blue square). Circled in red is BTV-5 positive in all three dogs. Only serotypes BTV 1-24 were tested. The base figure is replicated with permission and can be found in van Rijn PA, Maris-Veldhuis MA, Spedicato M, Savini G, and van Gennip RGP. Pentavalent disabled infectious single animal (DISA)/DIVA vaccine provides protection in sheep and cattle against different serotypes of bluetongue virus. *Vaccines*. 2021; 9(10):1150. https://doi.org/10.3390/vaccines9101150.

Antibodies to BTV were detected in four (67%) of the farm dogs with the cELISA assay. All four dogs testing cELISA positive had high levels of BTV antibodies on Day 14 and the levels remained high until Day 89 (Table 3). Dogs 1, 2, and 3 were tested with VN and all were positive for multiple serotypes. Serotypes with antibody titers >112 were serotypes BTV-3, BTV-5, BTV-6, BTV-16, BTV-20, and BTV-23. The only serotype with high titers (112) common to all three dogs was BTV-5 (Table 4 and Figure 2).

Viral isolation attempts were unsuccessful from samples from Dog 1 collected during clinical disease (*Ct* 24.7).

### 3.2. Sheep

Thirty-one percent (*n* = 5/16) of the sheep samples had detectable BTV RNA. In these sheep, the BTV RT-qPCR *Ct* values were 21.5, 22.0, 31.3, 38.0, and 38.2. Attempts to isolate the virus from samples collected from the sheep with the presumed highest viraemia (*Ct* 21.5) were unsuccessful.

### 3.3. *Culicoides* Midges

An estimated 61,000 midges were trapped and species identification was performed on 1/24th of the sample (*n* = 2,561 individual midges). The dominant species observed was *C. imicola* in 99.26% (*n* = 2,542 identified midges). Six other species were identified, namely *Culicoides leucosticus*, *Culicoides zuluensis*, *Culicoides enderleini*, *Culicoides subschultzei*, and *Culicoides neavei* (Table 5).

Four species of parous or gravid midges were detected: *C. imicola*, *C. leucosticus*, *C. zuluensis*, and *C. enderleini*. Of these, only batches of *C. imicola* were positive for BTV RNA using RT-qPCR, with 3/4 batches of 200 parous midges testing positive. Overall, midges were positive for sheep DNA in 90.3%, human DNA in 20.9%, horse DNA in 1.6%, and negative (DNA of tested species not detected) in 4.8% of blood-fed midges (Table 6).

## 4. Discussion

This study investigated the sheep farm of origin where a pregnant Rottweiler dog developed clinical BT disease. In addition to a surviving pregnant dog, natural infection with BTV RNA was detected in other farm dogs, sheep, and midges. Of the six study dogs, three (50%) had detectable BTV RNA and four (67%) had BTV-specific antibodies. If capable of onward transmission, the finding of naturally occurring BT viraemia in dogs suggests that dogs might potentially play a greater role in the introduction of BTV to disease-free areas than previously thought. Our study findings also support the previous studies that report *C. imicola* midges as the most important BTV vector in the inland areas of South Africa accounting for 99.3% of trapped midges and the only species from which BTV RNA was detected [7, 8, 32]. However, this study failed to determine midge feeding on dogs and the route of transmission remains unclear on this farm. The serotype responsible for this farm was not definitively determined. Further studies are needed regarding dogs and the route of natural BTV transmission, their potential role in transmission, and exposure frequency to quantify their potential role in BT epidemiology.

Despite the high prevalence of BTV on this farm, the only dog to show clinical signs was Dog 1 (a pregnant dog). All other dogs showed no abnormal clinical signs or behavior according to the owners before or during the observational period. Inclusive of this study, clinical BT disease has only occurred in pregnant dogs [14, 17, 33, 34]. The reason for this is unknown [33, 34, 35] but could be associated with the relative abundance of sialic acid cell receptor sites used by BTV for attachment and entry into host cells [36]. These receptor sites appear to be upregulated during pregnancy in other species [37] and investigation of this phenomenon in dogs could be worthwhile. Pregnant dogs with unexplained respiratory disease, abortion, or death should be investigated for the possibility of BTV infection in areas where the disease occurs [21].

Dog 1 presumably had a high viraemic level (*Ct* 24.7) during clinical disease (Day 3). Samples collected from two apparently healthy dogs (Dogs 2 and 3) with BTV nucleic acid detected presumedly had lower viraemic levels as evidenced by the higher *Ct* values of 36.0 and 39.1 (classified as weak positive). All dogs with detectable nucleic acid were confirmed to have been exposed to BTV with high antibody titers using both cELISA (PI > 85) and VN (>112) tests. Samples collected at the first farm (Day 14) visit from the subclinical dogs (Dogs 2 and 3) had both detectable BTV RNA and antibodies to BTV. Previous BTV experimental exposure studies showed that the earliest BTV antibody response in exposed dogs occurred from 7 to 10 days postexposure with cELISA (>50% PI) [16]. In these exposure studies, the rising cELISA PI values coincided with waning viral loads based on viral isolation [16]. Based on these studies, it is reasonable to assume that the dogs in our study were exposed at least a week before sampling and the peak BT viraemia levels likely occurred before the first blood collection. This peak viraemia was likely to be greater (lower *Ct* values) than when tested, but it is not known if this peak viraemia occurred at levels sufficient for BTV transmission.

Despite the low viraemic levels, the detection of the BTV nucleic acid in subclinical dogs is of substantial interest because of the potential this holds for BTV introduction. Bluetongue viraemia in dogs without clinical signs greatly increases the potential of BTV introduction through the importation of apparently healthy dogs [12, 24, 38]. This role might be significant given the high BTV seroprevalence in African dogs reported in previous studies (20%–21%) [12, 24]. Dogs are frequently exported from BT endemic areas [39, 40] but there are few restrictions relating to BTV for the species [3, 41]. The importation of clinically ill dogs infected with BTV would be exceedingly unlikely given the severity of the disease [21, 24, 33]. Due to the high exposure in African countries, the importation of subclinical but viraemic dogs was previously suggested as a possible source of BT outbreaks in Europe where the source of infection was not determined [12, 24].

The duration of viraemia could not be determined in farm dogs without knowledge of when initial exposure occurred but appeared to be short-lived. The clinically ill dog (Dog 1) had high levels of BTV RNA during clinical illness (Day 3); not detected 10 days later (D14); then low levels of RNA were detected on Day 28 and again not detected on Day 60. This lack of detection on Day 14 samples could represent the limit of detection with low levels of BTV RNA and repeated testing of D14 samples might have been interesting but was not performed. For the subclinical positive dogs (Dogs 2 and 3) samples tested positive for BTV RNA on only the first blood collection (Day 14) but not detected in any subsequent samples (on Days 28 and 60). Previous experimental studies reported a short-lived viraemia in infected dogs that were undetectable 10 and 14 days after exposure, using virus isolation and RT-qPCR, respectively [16, 23].

The current study shows that the duration of BTV detection with RT-qPCR in dogs was substantially shorter than seen in ruminants. Ruminants remain positive for BTV RNA using the RT-qPCR assay for up to 5 months following infection [42]. However, the duration of infectious viraemia in sheep and wild ruminants is reported to much shorter (<11 days) and cattle can be infectious for as long as 9 weeks [43, 44, 45]. Positive RT-qPCR testing is not associated with the duration of infectious viraemia but rather with the presence of nonviable and cell membrane-bound viral particles [42]. The reason BTV RNA detection with RT-qPCR detection was so short in dogs is unknown but likely relates to quicker clearance of infected cells and cell-associated nonviable viral particles in the species. A short BT viraemic period in dogs markedly diminishes the potential role of dogs in ongoing vector transmission and disease introduction to disease-free areas.

Concerning the mode of transmission, all dogs with detectable BTV nucleic acid had direct contact with sheep. The dogs had not received any recent vaccinaton and deliberate feeding of sheep meat did not occur on this farm. Transmission to dogs in contact with sheep could have been through ingestion of afterbirth or aborted material, through direct contact or vector-borne. Oral BTV transmission has occurred through the ingestion of colostrum in ruminants [46] and the ingestion of BTV-contaminated meat in two lynxes [13]. Direct contact transmission has been suggested to occur in goats infected with BTV-26 [47]. The most recent report of BT in dogs was in the Netherlands, 2023, where a pregnant dog was reported to develop illness during the BTV-3 outbreak [22]. This case supports previous reports of dogs developing BT disease through natural transmission [18, 22]. The route of transmission in a pregnant dog from the Netherlands was also suspected to be either through ingestion of afterbirth or through midges and similarly could not be determined definitively [22]. One of the seropositive dogs (Dog 6) had no direct contact with sheep or the RT-qPCR-positive dogs. If this dog was exposed during the flock investigation, vector-borne transmission was probable but cannot be confirmed. Further evidence of this transmission route could not be collected based on the employed study design. Midge-borne transmission of BTV to dogs has been previously suspected in Morocco [38].

Remarkably, BTV infection in this sheep flock was only detected as a direct result of an initial BT diagnosis in a dog. Detection of BT in this sheep flock allowed for the implementation of measures aimed at reducing sheep mortalities that were occurring on this farm. This was similar to a recent report of BTV isolation from a sick pregnant dog that resulted in the diagnosis of BTV-3 in a cattle herd in the Netherlands [22]. This report and the current study motivate for the inclusion of dogs in BT surveillance. The prevalence of detectable BT viraemia in dogs and sheep on this farm was similar (*n* = 2/6 dogs and *n* = 5/16 sheep) based on the RT-qPCR assay on samples collected on the first (Day 14) farm visit. Dogs are abundant and ubiquitous in all areas of human settlement [48] and might also be easier to sample than wild ruminants. As such, testing of dogs might offer a useful addition to BT surveillance in certain scenarios such as areas where vaccines are administered (as dogs as not vaccinated against BTV) and where sampling of wild or domestic ruminants is difficult.

Canine blood meals were not detected in any midge sample tested. Midge feeding on dogs is thought to be rare but factors influencing this are poorly understood [38, 49, 50, 51]. Dog blood meals have been previously detected in several midge species including *C. imicola* [31, 52, 53, 54, 55]. The absence of canine blood meal detection does not exclude vector-borne BTV transmission to dogs occurrence on this farm for the following reasons. Midge feeding is influenced by the abundance of host body mass in an area and on this farm, dogs represented less than 3% of domestic mammals on this farm potentially limiting the detection of midge feeding on dogs [56, 57]. The trap was placed directly above the sheep due to available electrical points and midges feeding on dogs might not have reached this trap. Farm dogs had also received the ectoparasitic drug fluralaner that might also have caused high midge mortality after feeding on their blood as has been described for sandflies and mosquitoes [58, 59]. The absence of detection of dog blood might also highlight a potential limitation of using trapped midges for identification of mammalian host species of BTV with relatively small population sizes in trapping areas.

Regarding the other mammalian host species detected, *C. imicola* has a high affinity for human blood meals found in up to 78% of blood-fed midges tested in previous studies [55]. The presence of human DNA detected (*n* = 13/62) could also be in part due to the contamination of samples during testing and sample preparation and high analytical test sensitivity. Three blood-fed midge samples were negative (*n* = 3/62) for the domestic species DNA tested and might have fed on other wildlife species or birds (not tested) or contained blood meals too degraded for detection as reported in other studies [31, 60].

The BTV serotype responsible for clinical disease on this farm could not be definitively determined and is a shortcoming of this study. Serotype determination was expected to be achieved through plaque inhibition from viral isolates. When virus was not isolated, the investigators attempted to determine the BTV serotype by examining VN serotype-specific antibody titers. This also failed to definitively identify the implicated serotype. High serotype BTV-5 antibody titers were common in all VN serologically tested dogs but each dog had high VN titers to several other serotypes complicating the identification of the BTV serotype. Antibodies to multiple BTV serotypes could have been due to the production of nonserotype-specific or cross-reactive BTV antibodies in these dogs. The pattern of this potential cross-reactivity observed in dogs differed from known serotype cross-reactivity observed in ruminants receiving modified live vaccines (Figure 2) [61]. Bluetongue serotype-specific RT-qPCR would likely have identified the serotype but was not readily available and samples collected from Dog 1 during clinical illness were exhausted during viral isolation attempts.

Failure to isolate virus from samples collected during the clinical illness of Dog 1 (*Ct* 24.7) was likely related to the erroneous sample storage at −20°C for a prolonged period. Bluetongue virus is reported to be unstable at −20°C but remains viable for prolonged periods at −70°C and 4°C [3]. This is supported by the unsuccessful viral isolation from a sheep also with a presumed high viraemia (Ct 21.5) stored in similar conditions. In addition, isolation attempts were only made using BSR-T7 cell lines, a clone of the baby hamster kidney-derived (BHK) cell lines that have both been used successfully for BTV isolation [27, 62]. Successful isolation might have been achieved on embryonated chicken eggs or an insect cell line as suggested by the WOAH manual [26], but this was not performed. Finally, the potential presence of BTV antibodies in samples might also have prevented successful isolation.

This was a field investigation involving a few animals with the potential for unknown factors being uniquely specific to this farm that limit inferences to other populations. Some of the laboratory methods employed have not been validated for use in dogs but have been previously used in dogs [12, 18, 24] and are not species-specific [26]. Only *Culicoides* midges were sampled in this study and while less likely than midge-borne transmission, an unidentified alternative vector (such as a tick or mosquito) could have been involved in virus transmission to farm dogs. Despite these limitations, this study showed natural BTV exposure and infection can occur in dogs on farms experiencing BT infection in sheep.

## 5. Conclusion

On this farm, dogs that were naturally exposed to BTV developed viraemia and had detectable humoral immune responses even in animals without clinical disease. Naturally occurring viraemia, especially in subclinical or dogs with only mild clinical signs, is an important finding as viraemic dogs could be imported from areas experiencing BT infection in sheep into disease-free countries or areas. As the detection of BTV nucleic acid appeared short-lived and no definitive evidence of midge-borne transmission to or from dogs was found, the species transmission potential might be limited. However, the inclusion of dogs in BT surveillance programs and research programs is recommended to assess the transmission potential and significance of this atypical BTV host species.

## Figures and Tables

**Table 1:** Details of the six dogs (designated Dogs 1–6) living on a sheep farm with BT infections.

Dog	Sex	Breed	Age (years)	Pregnant	Farm area	Direct contact with sheep
1^a^	FI	Rottweiler	3.5	Yes	Sheep kraal	Yes
2	MI	Rottweiler	3.5	No	Sheep kraal	Yes
3	FS	Medium-sized mixed	6.0	No	Sheep kraal	Yes
4	FS	Rottweiler	7.0	No	Inside and outside the house	No
5	MI	Rottweiler	8.0	No	Camp A, outside the house	No
6	FI	Rottweiler	2.5	No	Camp A, outside the house	No

The table displays the sex, breed, age, sterilization status, the location where the dogs were kept, and whether the dogs had direct contact with sheep. ^a^Dog 1 was pregnant, and the first BT case was diagnosed on the farm; FI, female intact; MI, male intact; FS, female sterilized.

**Table 2:** Results of the BTV RT-qPCR assay threshold cycle (*Ct*) of samples collected from dogs on this farm.

Farm dogs	BTV RT-qPCR *Ct* values			
	Day 3 (clinical disease)	Day 14	Day 28	Day 60
Dog 1^a^	24.7	nd	38.2	nd
Dog 2	—	36.0	nd	nd
Dog 3	—	39.0	nd	nd
Dog 4	—	nd	—	—
Dog 5	—	nd	—	—
Dog 6	—	nd	—	—

BTV RT-qPCR, bluetongue real-time reverse transcriptase polymerase chain reaction; nd, BTV RNA not detected (*Ct* > 40). Days after the onset of clinical disease in Dog 1. ^a^Dog 1 was the only dog showing clinical signs of BT.

**Table 3:** Result of serological testing for BTV antibodies using cELISA (percentage inhibition) and VN (maximal titers) of samples collected from farm dogs.

Farm dogs	BTV cELISA PI				VN titer (Table 4)
	Day 14	Day 28	Day 60	Day 89	
Dog 1	85	85	86	89	>360
Dog 2	89	90	84	79	>360
Dog 3	87	85	76	80	112
Dog 4	nd	(nt)^a^	(nt)^a^	nd	(nt)
Dog 5^a^	nd	(nt)^a^	(nt)^a^	nd	(nt)
Dog 6^a^	90	(nt)^a^	(nt)^a^	73	(nt)

SNT, serum viral neutralization; cELISA, BTV VP7 protein iGG blocking competitive enzyme-linked immunosorbent assay; PI, percentage inhibition of control serum (100 − ((mean absorbance test sample)/(mean absorbance MAb control) × 100); nd, not detected; (nt), not tested. ^a^Dogs not tested at Day 28 and Day 60 due to aggression and negative RT-qPCR result from samples collected on Day 14.

**Table 4:** The results of BTV VN (maximal titers) for the serotypes BTV-1 to BTV-24 of samples collected from three dogs where BTV RNA was detected on RT-qPCR.

BTV serotype	Dog 1 (*Ct* 24.7)	Dog 2 (*Ct* 36.0)		Dog 3 (*Ct* 39.0)		Median SNT dilutions (range)
	Day 28^a^	Day 28	Day 60	Day 14^b^	Day 60	
BTV-1	28	28	28	(nt)	0	28 (0, 28)
**BTV-2**	0	**112**	**224**	(nt)	0	56 (0, 224)
**BTV-3**	**>320**	**>320**	**>320**	(nt)	0	**>320 (0, 340)**
BTV-4	56	80	160	(nt)	0	68 (0, 160)
**BTV-5**	**>320**	**>320**	**>320**	(nt)	**112**	**>320 (112, >320)**
**BTV-6**	**320**	(nt)	28	(nt)	56	56 (28, 320)
BTV-7	40	28	28	(nt)	0	28 (0, 40)
BTV-8	10	(nt)	0	(nt)	0	0 (0, 10)
**BTV-9**	**320**	(nt)	28	(nt)	0	28 (0, 320)
**BTV-10**	**160**	(nt)	0	40	(nt)	40 (0, 160)
BTV-11	14	80	80	(nt)	0	47 (0, 80
**BTV-12**	28	**224**	**320**	(nt)	**0**	**126 (0, 320)**
BTV-13	>320	28	28	(nt)	0	28 (0, >320)
BTV-14	0	(nt)	14	(nt)	0	0 (0, 14)
**BTV-15**	**224**	28	28	(nt)	**112**	70 (28, 224)
**BTV-16**	**320**	**112**	**112**	(nt)	0	**112 (0, 320)**
**BTV-17**	**112**	(nt)	0	(nt)	0	0 (0, 112)
BTV-18	0	(nt)	0	(nt)	0	0 (0, 0)
BTV-19	56	28	14	(nt)	14	21 (14, 56)
**BTV-20**	**320**	(nt)	80	80	(nt)	80 (80, 320)
BTV-21	56	(nt)	0	0	(nt)	0 (0, 56)
BTV-22	20	(nt)	40	(nt)	80	40 (20, 80)
**BTV-23**	**320**	(nt)	40	40	(nt)	40 (40, 320)
**BTV-24**	**112**	28	—	(nt)	0	42 (0, 112)
Median (IQR)	84 (22, 320)	28 (14, 104)	80 (28, 168)	40 (10, 70)	0 (0, 11)	28 (0, 112)
Median per dog (IQR)	84 (22, 320)	—	28 (0, 84)	—	0 (0, 40)	—

Titers greater than 112 are highlighted in bold. BTV, bluetongue virus; SNT, serum viral neutralization titers; IQR, interquartile range, (nt), not tested. ^a^Due to laboratory issues, samples collected on Day 60 from Dog 1 were exhausted without results and testing was only performed on Day 28 samples; ^b^due to inadequate volume BTV-10, -20, -21, and -23 were tested on samples collected Day 14 from Dog 3.

**Table 5:** Species, sex, and reproductive stage of *Culicoides* midges collected over 36 hr using an Onderstepoort 12 V light trap on a farm during a bluetongue outbreak involving dogs.

Midge species	Female			Male	Total (%)	BTV RT-PCR (parous midges)
	Nulliparous	Parous	Blood fed			
*C. imicola*	936	1,527	34	45	2,542 (99.34)	Positive^a^
*C. leucosticus*	1	1	—	—	2 (0.08)	Negative^b^
*C. zuluensis*	4	3	—	—	7 (0.27)	Negative^b^
*C. enderleini*	2	2	—	—	4 (0.16)	Negative^b^
*C. subschultzei*	—	—	—	3	3 (0.12)	—
*C. neavei*	—	—	—	1	1 (0.04)	—
Subsample total (%)	943 (36.9)	153 (59.9)	34 (1.3)	49 (1.9)	2,559	—
Estimated total sample collected (subsample × 24)					61,416	—

BTV RT-qPCR, bluetongue real-time reverse transcriptase polymerase chain reaction. ^a^Tested in four batches of 200 parous midges, three tested positive (*Ct* values of 25.6, 36.8 and 35.7); ^b^all parous tested in one batch.

**Table 6:** The results of DNA speciation probes in blood-fed Culicoides midges trapped on a farm, 14 days after the appearance of clinical signs in Dog 1.

		Individual midges	Batches of 10 midges	Batches of unsorted midges	Total (% of tests)
Midge species and numbers tested	Midge species	C. *imicola*	*C. imicola*	Not sorted	—
	Midges per test	1	10	34^a^	—
	Number of tests	40	21	2	63
	Blood-fed midges tested	40	210	68	318

Species of domestic animals' DNA detected in blood-fed midges	Sheep	36 (90.0%)^b^	18 (90.0%)^c^	2 (100%)	56 (90.3%)
	Human^d^	10 (25.0%)^b^	3 (15.0%)^c^	0	13 (21.0%)
	Horse	0	1 (5.0%)	0	1 (1.6%)
	Cat	0	0	0	—
	Cow	0	0	0	—
	Dog	0	0	0	—
	Donkey	0	0	0	—
	Goat	0	0	0	—
	Pig	0	0	0	—
	None detected	4 (10.0%)	1 (5.0%)	0	5 (4.6%)

Midges were trapped for 36 hr using an Onderstepoort 12 V UV light trap run above sick sheep, lambs, and periparous ewes housed in an observation pen. ^a^Estimated number of blood-fed midges in 1/24th of the total sample (2,559 midges); ^b^midges were positive for human and sheep blood 20% (*n* = 8); ^c^midge batches were positive for human and sheep blood in 15% (*n* = 3); ^d^the presence of human DNA is assumed could occur with laboratory contamination.

## Data Availability

All data and additional information will be made available upon request and uploaded onto the University of Pretoria's data repository.
